# Analysis of clinical outcomes of intra-aortic balloon pump during coronary artery bypass surgery

**Published:** 2015

**Authors:** Gunduz Yumun, Ufuk Aydin, Yusuf Ata, Faruk Toktaş, Arda Aybars Pala, Ahmet Fatih Ozyazicioglu, Tamer Turk, Senol Yavuz

**Affiliations:** Department of Cardiovascular Surgery, Namik Kemal University, Tekirdag, Turkey; Department of Cardiovascular Surgery, Bursa Yuksek Ihtisas Education and Research Hospital, Bursa, Turkey; Department of Cardiovascular Surgery, Bursa Yuksek Ihtisas Education and Research Hospital, Bursa, Turkey; Department of Cardiovascular Surgery, Bursa Yuksek Ihtisas Education and Research Hospital, Bursa, Turkey; Department of Cardiovascular Surgery, Bursa Yuksek Ihtisas Education and Research Hospital, Bursa, Turkey; Department of Cardiovascular Surgery, Bursa Yuksek Ihtisas Education and Research Hospital, Bursa, Turkey; Department of Cardiovascular Surgery, Bursa Yuksek Ihtisas Education and Research Hospital, Bursa, Turkey; Department of Cardiovascular Surgery, Bursa Yuksek Ihtisas Education and Research Hospital, Bursa, Turkey

**Keywords:** intra-aortic balloon pump, coronary artery bypass, mortality

## Abstract

**Aim:**

The mortality rate in coronary artery bypass surgery increases with advancing patient age. This study was conducted to analyse and compare older (above 65 years of age) with younger patients (below 65 years of age) who had undergone coronary artery bypass surgery and had an intra-aortic balloon pump (IABP) inserted, comparing hospital stay, clinical features, intensive care unit stay, postoperative complications, and morbidity and mortality rates.

**Methods:**

One hundred and ninety patients who had undergone coronary artery bypass surgery and required IABP support were enrolled in this study. Patients younger than 65 years of age were considered young, and the others were considered old. Ninety-two patients were young and 98 were old. The mortality rates, pre-operative clinical characteristics, postoperative complications, and duration of intensive care unit and hospital stays of the groups were compared. The risk factors for mortality and complications were analysed.

**Results:**

One hundred and thirty-eight of the patients were male, and the mean patient age was 62.7 ± 9.9 years. The mortality rate was higher in the older patient group than the younger group [34 (37.7%) and 23 (23.4 %), respectively (*p* = 0.043)]. The cross-clamp time, mean ejection fraction, cardiopulmonary bypass time, and length of stay in the intensive care unit were similar among the groups (*p* > 0.05). Cardiopulmonary bypass time was the single independent risk factor for mortality in both groups.

**Conclusion:**

In this study, high mortality rates in the postoperative period were similar to prior studies regarding IABP support. The complication rates were higher in the older patient group. Prolonged cardiopulmonary bypass and advanced age were determined to be significant risk factors for mortality.

## Abstract

An intra-aortic balloon pump (IABP) increases coronary blood flow and reduces left ventricular afterload.^1^-^3^ It helps to increase the necessary amount of time for heart recovery in low cardiac output syndrome following a cardiopulmonary bypass (CPB) or ischaemic events. In earlier reports, researchers had suggested that postoperative heart failure was the single indication for IABP support.^1^,^2^ However, these indications have widened, and the use of IABP support has recently become more common.

Frequently reported complications of IABP include bleeding, aorto-iliac injury and thrombocytopenia.^4^,^5^ In-hospital mortality and early mortality of patients requiring IABP support is high, ranging from 26 to 50%, due to the cardiac problems that initially led to the need for this support.^6^,^7^

The elderly population is continuously increasing across the globe. Parallel with this increase, the number of older patients being referred for coronary artery bypass grafting (CABG) has also increased.^8^ Although several studies have shown a significant increase in surgical mortality of elderly patients,^9^ there have been no studies regarding clinical outcomes of IABP in elderly patients.

In the present study, we aimed to analyse and compare older with younger patients, regarding clinical features, postoperative complications, intensive care unit and hospital stays, and morbidity and mortality rates in patients who had undergone CABG surgery and required IABP support.

## Methods

Patients who had undergone CABG in our clinic between 2008 and 2013 were retrospectively evaluated. Patients who had undergone combined CABG and heart valve surgery were excluded. This study was granted the full approval of the institutional review board.

Three hundred and eighty-eight (7.4%) of 4 940 consecutive patients required IABP support following CABG. Among these patients, IABP was used intra-operatively for 190 patients. One hundred and thirty-eight of the patients were male, and the mean patient age was 62.7 ± 9.9 years. The demographic characteristics of the patients are summarised in Table 1.

**Table 1:** Demographic characteristics of the patients

	*Younger group (n = 98)*	*Older group (n = 92)*	*Total*	*p-value*
Gender (M:F)	74/24	64/28	138/52	0.358
Mean age (years)	54.7 ± 6.1	71.4 ± 4.5	62.7 ± 9.9	< 0.001
Mean EF (%)	37.1 ± 8.3	39.2 ± 9.5	38.1 ± 8.9	0.121
MI, n (%)	31 (31.9)	24 (26)	55 (27.7)	0.400
COPD, n (%)	5 (5.1)	13 (14.1)	18 (9)	0.034
CRF, n (%)	3 (3)	5 (5.4)	8 (4.2)	0.487
Redo, n (%)	3 (3)	0	3 (1.5)	0.297
HT, n (%)	47 (48)	56 (60)	103 (54)	0.074
DM, n (%)	47 (48)	23 (25)	71 (37.3)	0.001
CVA, n (%)	4 (4.1)	5 (5.4)	9 (4.7)	0.745
Recent MI, n (%)	18 (18.3)	16 (17.4)	34 (17.9)	0.861
EuroSCORE	4 (0–10)	5 (2–10)	4 (0–10)	< 0.001
BMI (kg/m2)	27.2 ± 4	26.7 ± 4.4	27.2 ± 4.1	0.112
LMCA, n (%)	8 (8.1)	5 (5.4)	13 (6.8)	0.457
Prophylactic levosimendan, n (%)	18 (18.3)	12 (13)	30 (15.8)	0.315
Emergency, n (%)	18 (18.3)	16 (17.4)	34 (17.8)	0.861
Pre-operative IABP, n (%)	8 (8.1)	9 (9.7)	17	0.405

COPD: chronic obstructive pulmonary disease, CRF: chronic renal failure, HT: hypertension, DM: diabetes mellitus, CVA: cerebrovascular accident, BMI: body mass index, LMCA: left main coronary artery disease, EF: ejection fraction.

All of the patients were operated on with standard CPB under general anaesthesia. Antegrade cardioplegia was used for cardiac protection. In all cases, an IABP catheter was inserted through the common femoral artery.

In this study, IABP was used intra-operatively when weaning from CPB failed. Pre-operative IABP was used in cases of low cardiac output, unstable refractory angina, or persistent arrhythmia due to myocardial ischaemia.^5^,^10^

The patients were classified according to their ages: whether they were younger than 65 years or older. The mortality rate, complications of IABP, intra-operative properties, pre-operative clinical characteristics of patients, and length of stay in the intensive care unit (ICU) were recorded.

The pre-operative parameters of the patients were age, gender, re-operation, hypertension, body mass index, diabetes mellitus, chronic renal failure, the value of the EuroSCORE, previous cerebrovascular accidents, left ventricular ejection fraction, left main coronary artery disease, chronic obstructive pulmonary disease (COPD), and the presence of a myocardial infarction more recently than one week previously. The pre-operative clinical characteristics, postoperative complications, duration of ICU and hospital stays, and mortality rates of the groups were compared.

## Statistical analysis

Demographic characteristics were compared using mean and median values. Parametric results were evaluated using the Student’s *t*-test and Tukey test. The chi-square method, Pearson’s test, and Fisher’s test were used to analyse the categorical parameters. Risk factors for mortality were assessed using a binary logistic regression analysis. The standard deviation value of *p* < 0.05 was considered significant. SPSS 18 was used for the statistical analysis.

## Results

In this study, 138 of the 190 patients were male. The mean patient age was 62.7 ± 9.9 years. Ninety-eight patients were younger than 65 years of age, and 90 patients were 65 years of age or older.

The number of patients with COPD and the mean EuroSCORE of the patients were higher in the older group. In contrast, the number of patients with diabetes mellitus was higher in the younger group. In terms of other demographic characteristics, there were no statistically significant differences between the groups (Table 1). The mean CPB times, mean crossclamp times, and number of grafts used were similar between the two groups (Table 2).

**Table 2:** Mortality rates and clinical outcomes of the patients

	*Younger group*	*Older group*	*p-value*
Mortality, n (%)	23 (23.4)	34 (36.9)	0.043
Mortality, n (%)*	8 (44.4)	7 (41.1)	0.964
Mortality, n (%)**	15 (18.7)	27 (36)	0.018
CPB time (min)	143 ± 59	140 ± 58	0.786
Graft number	3.1 (2–5)	3.2 (2–5)	0.789
Cross-clamp time (min)	90 ± 34	88 ± 38	0.604
ICU time (day)	5.9 ± 4	6.6 ± 5	0.284

ICU: intensive care unit. CPB time: cardiopulmonary bypass time, *patients with emergency operation, **patients undergoing elective operation.

Fifty-seven (30.1%) patients died in the first 30 days following the operation. Twenty-three of these patients were in the younger group. The mortality rate of the younger group was significantly lower compared to the older patients (*p* = 0.043). In the subgroup analysis, the mortality rate of emergent operations was similar in the both groups (*p* = 0.964). However, the mortality rate was higher in the older group for elective operations (*p* = 0.018).

Among the surviving patients, the number of older patients, rate of emergency operations, mean EuroSCORE values, and number of patients with chronic renal failure were lower than that in the group of patients who died (Table 3). Binary logistic regression analysis showed that the only factor affecting mortality was prolonged CPB time. However, in the subgroup analysis of patients without emergency conditions, age was the second determinant of mortality (*p* = 0.018, OR = 5.5).

**Table 3:** Parameters of patients who survived or died

	*Patients who survived (n = 133)*	*Patients who died (n = 57)*	*p-value*
Pre-operative MI, n (%)	40 (30)	15 (26.3)	0.601
BMI (kg/m2)	27.5 ± 4.2	26.9 ± 4	0.507
EuroSCORE	4.2 (0–10)	5.1 (0-10)	0.030
DM, n (%)	47 (35.3)	24 (42.1)	0.377
CRF, n (%)	3 (2.2)	5 (8.7)	0.040
Mean EF (%)	38.4 ± 8	37.5 ± 9	0.562
Mean age (years)	61.8 ± 9.8	64.9 ± 10	0.051
Older patients, n (%)	58 (43.6)	34 (59.6)	0.043
Gender (M:F)	33/101	20/37	0.118
COPD, n (%)	12 (9)	6 (10.5)	0.746
Emergency operation, n (%)	19 (14.2)	15 (26.3)	0.047
LMCA, n (%)	8 (6)	5 (8.7)	0.490
CVA, n (%)	5 (3.7)	4 (7)	0.333
HT, n (%)	69 (51.8)	34 (59.6)	0.328
Re-operation, n (%)	3 (2.2)	0	0.555
Pre-operative IABP, n (%)	14 (10.5)	3(2.2)	0.405
CPB time (min)	130 ± 48	167 ± 72	< 0.001
Cross-clamp time (min)	87 ± 35	94 ± 36	0.180

CPB time: cardiopulmonary bypass time, COPD: chronic obstructive pulmonary disease, CRF: chronic renal failure, HT: hypertension, DM: diabetes mellitus, CVA: previous cerebrovascular accident, BMI: body mass index, LMCA: left main coronary artery disease.

In the subgroup analysis, CPB time and pre-operative chronic renal failure were independent risk factors for mortality in the older group. In the younger group, female gender, diabetes mellitus, high EuroSCOREs, emergency operations, prolonged CPB (*p* = 0.001, OR = 7.6), and prolonged stays in the ICU were independent risk factors for mortality (Table 4).

**Table 4:** Factors for mortality in subgroup analysis

	*Younger group*		*Older group*	
	*Odds ratio*	*p-value*	*Odds ratio*	*p-value*
COBD	0.035	0.851	0.015	0.903
CRF	0.168	0.682	4.205	0.040
Re-operation	0.949	0.330	-	-
EF (%)	0.865	0.352	0.110	0.759
Age (years)	0.122	0.727	1.034	0.741
EuroSCORE	14.555	0.000	8.418	0.309
CPB time (min)	7.698	0.006	0.471	0.004
Cross-clamp time (min)	2.048	0.152	1.542	1.542
BMI (kg/m2)	0.703	0.402	0.384	0.214
Emergency operation	5.401	0.020	0.400	0.536
Female gender	8.850	0.003	1.725	0.527
HT	2.007	0.157	0.095	0.189
MI	0.427	0.513	0.004	0.758
DM	7.477	0.006	0.560	0.949
ICU time	4.947	0.026	0.038	0.454
Levosimendan	0.228	0.633	0.131	0.845
CVA	1.634	0.201	0.021	0.717
LMCA	0.955	0.329	0.021	0.885

CPB time: cardiopulmonary bypass time, COPD: chronic obstructive pulmonary disease, CRF: chronic renal failure, HT: hypertension, DM: diabetes mellitus, ICU: intensive care unit, CVA: previous cerebrovascular accident, BMI: body mass index, LMCA: left main coronary artery disease.

In our study, a few serious complications were observed due to IABP support. Iliac artery injury occurred in two patients and peripheral ischaemia was observed in three patients. The other complications were thrombocytopenia and minor bleeding at the catheter site (Table 5). The rate of complications was similar between the groups.

**Table 5:** IABP complications according to patient group

	*Younger group*	*Older group*	*p-value*
Bleeding, n (%)	1 (1)	4 (4.3)	0.200
Arterial injury, n (%)	0	2 (2.1)	0.233
Mild thrombocytopenia, n (%)	10 (10.2)	15 (16.3)	0.309
Extremity ischaemia, n (%)	1 (1)	2 (2.1)	0.611
Total, n (%)	12 (12.2)	23 (25)	0.023

## Discussion

Postoperative recovery in elderly patients requires a longer time period than for younger patients. Postoperative atrial fibrillation requiring medical treatment, and other complications occur more frequently in the elderly; the total intubation time was also longer for this group. Therefore, delayed recovery in the elderly may simply be due to the aging process affecting all organs.^9^ For this reason, elderly patients may need more mechanical support in cases of low cardiac output following CPB.

In the present study, while the number of COPD patients was higher in the older group, the number of diabetes mellitus patients was lower than in the younger group. In addition, EuroSCORE values were higher in elderly patients. The mortality rate was higher in elderly patients; however, there were no statistically significant differences between the patients who had emergency surgery in both groups.

It has been reported that IABP decreases the mortality rates of low cardiac output and severe myocardial ischaemia patients in the pre-operative period, provides support for patients who failed to wean from CPB during the intra-operative period, and prevents low cardiac output and medically refractory arrhythmias in ICU in the postoperative period.^11^,^12^ In this study, IABP was used in cases of low cardiac output, persistent angina pectoris, or arrhythmia due to myocardial ischaemia in the pre-operative period.

In previous studies, the use of pre-operative IABP in high-risk patients was reportedly more advantageous than peri-operative IABP support. Böning *et al.* compared the use of pre-operative and peri-operative IABP in high-risk patients in their study. Their results indicate that the pre-operative use of IABP was advantageous for early and long-term mortality.^13^

Dyub *et al.* showed that in a meta-analysis involving 1 034 patients, the use of pre-operative IABP in high-risk patients reduced mortality.^14^ Holman *et al.* reported that when shock, urgent surgery, haemodynamic instability, and MI in the last three days were excluded, the use of pre-operative IABP did not have a positive effect on morbidity and mortality rates; however, the length of the hospital stay was less in these patients.^15^

Miceli *et al.* proposed a scoring system that predicts the need for IABP support in high-risk CABG patients.^16^ According to this study, heart failure, re-operations, emergency operations, left main coronary artery disease, patients over the age of 70 years, moderate and poor left ventricular function, and recent myocardial infarctions are independent risk factors for the need for IABP support. As a result of the study, the benefits of IABP support in patients with high-risk scores were emphasised. In our clinical practice, we did not use a risk-scoring system for prophylactic IABP support. In this study, we aimed to determine the pre-operative risk factors for mortality and other clinical outcomes.

In previous studies, emergency surgery, a history of myocardial infarction, prolonged CPB, and concomitant peripheral artery occlusive disease were all found to be significant determinants of mortality in primary isolated CABG patients.^17^ Furthermore, risk-scoring systems were generated. We showed that the mortality rate of the older patient group was higher than that of the younger group. However, the logistic regression analysis indicated that the only independent risk factor for mortality was a prolonged CPB time.

In addition, subgroup analysis revealed different results. For example, in the older patient group, chronic renal failure and prolonged CPB were identified as factors affecting mortality rate. In young patients, female gender, diabetes mellitus, emergency operations, higher EuroSCORE values, prolonged CPB, and prolonged stays in the ICU were independent risk factors for mortality. In elective operations advanced patient age and prolonged cardiopulmonary bypasses were identified as factors affecting mortality rates.

Complications with the IABP were described in previous studies: limb ischaemia, thrombocytopenia, arterial rupture or dissection, and sepsis and local infections.^4^–^6^,^10^,^18^ Complication rates have been reported from 26 to 50% in different studies. The risk factors for IABP complications were stated as increased age, female gender, duration of IABP treatment, presence of diabetes mellitus, and having several risk factors (e.g. obesity, smoking, hypertension, cardiogenic shock, inotropic support, low cardiac output, increased systemic vascular resistance, and ankle–brachial pressure index < 0.8).

In our study, the IABP complication rate was higher in older patients compared to younger ones (25 vs 12.2%). Mild thrombocytopenia was the most frequently detected complication. When thrombocytopenia is detected, IABP therapy is terminated immediately so that fewer bleeding complications occur.

Limitations: our study was a single-institution, retrospective study, which had a relatively small sample size. This research may require repeating in multicentres with randomised trials. Unaccounted for confounders may have been inherent in such a retrospective analysis.

## Conclusion

IABPs are important cardiac support instruments that are easily implemented and have beneficial effects for resolving transient ischaemic situations. Whether young or old, patients who require IABP support have a high risk of mortality. Moreover, elderly patients have increased incidences of co-morbid disease, which makes them even more at risk of death. We suggest that IABP might be used in the intra-operative period as a prophylactic device in elderly patients with multiple risk factors.
